# TRPM2 Channels: A Potential Therapeutic Target in Melanoma?

**DOI:** 10.3390/ijms241310437

**Published:** 2023-06-21

**Authors:** Hattie M. Foster, McKenzie N. Carle, Lukas R. Jira, David W. Koh

**Affiliations:** Department of Pharmaceutical & Biomedical Sciences, Ohio Northern University, Ada, OH 45810, USA; h-foster@onu.edu (H.M.F.); m-carle@onu.edu (M.N.C.); l-jira@onu.edu (L.R.J.)

**Keywords:** melanoma, skin cancer, TRPM2, multidrug resistance

## Abstract

The transient receptor potential, the melastatin (TRPM) subfamily, which consists of eight known members, appears to have significant importance in melanoma progression, treatment, and prognosis. As several members were originally cloned from cancerous tissue, initial studies aimed towards identifying TRPM involvement in cancer progression and tumorigenesis. For relevance in skin cancer, previous research has shown roles for several TRPM members in skin cancer progression, growth, and patient prognosis. One unique member, TRPM2, appears to have notable therapeutic potential in the treatment of melanoma. Previous and recent studies have demonstrated increased TRPM2 expression levels in melanoma, as well as important roles for TRPM2 in melanoma growth, proliferation, and survival. TRPM2 is thus an emerging target in the treatment of melanoma, where TRPM2 antagonism may offer an additional treatment option for melanoma patients in the future.

## 1. Introduction

Skin is the largest organ in the body and is responsible for many functions. Composed of the epidermis, dermis, and subcutaneous tissue, skin serves as a defensive barrier against harmful substances such as reactive oxygen species (ROS). Melanocytes are one cell type found in the epidermis that synthesizes melanin, utilized to protect the skin from solar damage. However, because of their cutaneous location and role in melanin synthesis, melanocytes are more susceptible to ROS [1]. Once protection against ROS is compromised, melanocytes become susceptible to the deleterious effects of oxidative stress. This can lead to disorders characterized by skin depigmentation, including melanoma [1]. Because melanoma is the most debilitating form of skin cancer, a greater understanding of its ability to continuously proliferate and metastasize is critical in order to eradicate these neoplasms.

## 2. TRPM Channels

Transient receptor potential melastatin (TRPM) channels have the distinction of being members of the largest family within the TRP superfamily of ion channels. They are non-selective cation channels involved in many processes in the body, including various sensory functions, cell proliferation, apoptosis, and detection of changes in osmolarity, redox, and pH [2,3,4]. A unique feature of this TRP subfamily is that several members are bi-functional ion channels, as they also contain functional enzymatic domains [3]. In mammals there are eight members of the TRPM subfamily presently known, with each member numbered in their chronological order of discovery. Four out of eight TRPM members were discovered to have a direct correlation to the regulation of melanocyte physiology and development of melanoma [3,4]. TRPM members 1, 2, 7, and 8 are currently being studied to assess and create novel treatments for aggressive melanoma. Their complete role in melanoma, which itself is a complex condition that involves many processes that drive its pathogenesis and progression, is not entirely understood. However, because of their emerging roles in melanoma, where they are hypothesized to be significant regulators in the process or cascade of events important for melanoma cell progression or survival [3], this review will primarily introduce TRPMs 1, 7, and 8, and focus on TRPM2. A summary of TRPM channels’ expression levels and possible functions in melanoma/skin cancer are shown in Table 1.

## 3. TRPM1 Channels

The function of TRPM1, the founding member of the TRPM subfamily, is not completely known. However, it has been shown to be localized primarily in retinal bipolar neurons and melanin-producing cells of the skin [5]. Within these cells, TRPM1 appears to have a critical role in the non-selective gating of cations. Among these cations is calcium, which mediates several of its physiological functions in normal cells. These functions include AKT activation, colony formation, cell mobility, and skin pigmentation [11,12]. This latter function forms the basis for the reason it has been targeted in studies involving skin pigmentation disorders. Further, it appears that TRPM1 is important for other physiological functions, including melanocyte growth, differentiation, and melanogenesis [3]. The pathophysiological roles of TRPM1 have been identified in skin injury leading to cancer. UVB has been shown to decrease TRPM1 levels, which in turn decreases calcium influx [13]. Because of this effect, loss of TRPM1 levels was shown to inversely correlate with damage levels incurred in skin cells [3]. Further, because TRPM1 was shown to exhibit tumor suppressor properties [4], decreased expression may serve as a biomarker for the development of a highly differentiated, aggressive form of melanoma since TRPM1 concentrations consistently decrease with the progression of the disease [3].

Within the gene encoding TRPM1, miR-211 was discovered in the sixth intron [14]. This unique microRNA was shown to play a key role in facilitating the tumor suppressor function of TRPM1 by inhibiting malignant melanoma progression [14]. Mechanistically, miR-211 was shown to regulate genes involved in melanoma, including KCNMA1, which has been linked to proliferation and metastasis in cancers, as well as insulin-like growth factor 2 (IGF2R) and transforming growth factor beta receptor II (TGFBR2), both important for facilitating the tumor suppressor function of TRPM1 [15]. This research subsequently led to the identification of miR-211 as a new potential target for further drug studies.

In summary, it appears that decreased levels of TRPM1 and miR-211 are observed in melanoma. Low levels of miR-211 are associated with highly aggressive metastatic malignant melanoma [15]. Mutations in this intron cause oncogenic properties resulting in pro-metastasis and invasive effects. Together, TRPM1 and miR-211 can serve as potential targets for treatment as they are key regulators with significant function in melanoma. Since TRPM1, as well as its alternative splicing variants (multiple transcripts have been identified in melanoma cells [5,8]), and miR-211 have all been shown to be involved somehow in the process of melanoma, current therapy to combat melanoma aims to identify and modulate these targets, since TRPM1 could be utilized as a biomarker of melanoma progression, and miR-211 levels or its protein targets could be supplemented using molecular biological techniques [3,4].

## 4. TRPM7 Channels

TRPM7 is one of the aforementioned “chanzymes” in the TRPM family, where its physiological role in normal cells is the non-selective gating of cations, as well as enzymatic activity that phosphorylates downstream substrates via its kinase domain [2]. It is theorized that this channel plays a role in detoxifying and protecting melanocytes [8]. In healthy, non-mutated cells, critical physiological functions include roles in embryogenesis via the development of melanocytes, skeleton, thymus, nervous system, kidney, exocrine pancreas, urinary bladder, and platelets, and also the regulation of magnesium entry into the cells [16]. In various cancer cells, TRPM7 was shown to positively regulate proliferation, progression, and cell invasion [16]. In melanoma, TRPM7 has been reported to be overexpressed in melanoma cell lines, but its function is not completely known in these cells [8]. Malignant melanoma has been shown to contain mutated TRPM7 that possesses oncogenic properties. Polymorphisms or mutations of TRPM7 have been reported in other types of cancer, but their functional significance in these cancers is not known [16]. This leads to regulation of survival, cell progression, growth, migration, invasion, dysregulated Mg^2+^ (as Mg^2+^ is known to positively facilitate cell cycle progression and DNA synthesis) and Ca^2+^, and epithelial–mesenchymal transition [16]. It is hypothesized that TRPM7 is required for tumor growth and progression due to supporting evidence that this channel is significantly overexpressed in aggressive carcinomas [16].

Chemical inhibitors of the TRPM7 channel inhibit proliferation, growth, migration, invasion, invadosome formation (highly complex molecular structures that facilitate extracellular matrix remodeling and invasion), and markers of EMT in cancer cells, showing potential for future treatment targets [16]. Agents shown to elicit TRPM7 inhibition include midazolam, ginsenosides, carvacrol, and xyloketal B [16]. Decreased Mg^2+^ gating appears to be an important event since cell cycle progression and cell growth is inhibited and the treatment effects can be reversed with Mg^2+^ supplementation. However, it is possible that inhibition of the kinase activity of TRPM7 contributes as well. Other studies show that TRPM7 has potential to be a biomarker in pre-malignant states, as it has been shown to be overexpressed in early stages of various cancers and also shown to contain genetic mutations/polymorphisms in cancers [16]. In summary, studies have shown several novel roles for TRPM7 in cancer, collectively establishing foundations for further research that may potentially benefit future melanoma patients via identifying, screening, preventing, and creating a prognosis. 

## 5. TRPM8 Channels

TRPM8 is a non-selective cation channel with a preference for gating calcium. It was discovered as the first TRP channel activated by temperature and subsequently shown to have physiological roles in sensory perceptions [17] in noncancerous cells. More specifically, TRPM8 can be activated at temperatures between 15 °C and 25 °C, which results in an increase in intracellular calcium levels [9]. In skin, TRPM8 can decrease pigment-producing activity in melanocytes by decreasing tyrosinase and tyrosinase-related protein-1 (TRP-1) expression levels [3]. For relevance in cancer, TRPM8—initially thought to be expressed from a prostate-specific gene—was subsequently found in several other cancers, including melanoma [18]. In melanoma cells, one study demonstrated that activation of TRPM8 caused calcium influx and decreased cell viability [19]. Mutated TRPM8 channels were identified in melanoma, which caused calcium channel dysregulation and subsequent downstream effects, including increased proliferation, uncontrolled growth, and dysfunctional apoptosis [9]. Taken together, initial studies have demonstrated that TRPM8 channels have several functions in cancer, including roles in cell proliferation, cell survival, and metastasis [9]. These functions appear to be unique from their roles in normal noncancerous cells, where activation of TRPM8 appears to have promising antitumor effects in cancer cells.

Due to its expression in melanoma, the TRPM8 channel appears to be a novel target in the treatment of melanoma. Viability of melanoma cells is decreased in a dose-dependent manner after treatment with a cold activating medication such as menthol, eucalyptol, or icilin [9]. The channel becomes activated through the shift in the voltage gated channel toward a more negative potential. In combination with a cooling agent, use of natural products or other ligands may be used as adjuvant therapy.

## 6. Other TRPM Channels

Other members of the TRPM family have fewer studies associated with cancer, or they have associated but not direct effects in melanoma. TRPM3 channels are temperature-sensitive cation channels that share significant sequence similarity with TRPM1 channels [4,20]. Melanoma-associated retinopathy (MAR) is a condition associated with malignant melanoma and believed to be caused by an autoimmune response to specific antigens. Autoantibodies were found to recognize both TRPM3 and TRPM1 in MAR [21], indicating a possible role as a biomarker or in the pathogenesis of MAR. The possibility of a role as a biomarker in MAR by TRPM family members could provide patient benefits by allowing early detection of this deleterious condition.

TRPM4 is a calcium-impermeable channel activated by intracellular calcium that may play a large role in uveal melanoma (UVM) [22]. The levels found in UVM are positively correlated with poor prognosis and are a consistent biomarker for prognosis. Higher expression levels of TRPM4 in UVM were significantly associated with pro-cancer signaling pathways, such as the P13K-AKT-mTOR signaling pathway; hypoxia; p53 pathway; TGFβ signaling pathway; G2M checkpoint; angiogenesis; proliferation; inflammation; and inflammatory response [22]. Inhibition of the PI3K/AKT/mTOR pathway was demonstrated to have favorable therapeutic potential as a target for the prevention and treatment of uveal melanoma [22].

TRPM5, which shares significant sequence similarity with TRPM4 [4], is an intracellular Ca^2+^-dependent monovalent cation channel that is associated with acidic pH signaling and induction of matrix metalloproteinase-9 (MMP-9) expression [7]. MMP-9 is a proteolytic oncogenic protein that plays a role in tumor formation [23]. Increased induction leads to migration, EMT, and survival of cancer cells. TRPM5 expression is closely associated with prognosis in patients with malignant melanoma as increased levels are indicative of poor prognosis and highly aggressive melanoma [7].

## 7. TRPM2 Channels

As many studies have investigated TRPM cation channels in a variety of cancer processes, including proliferation and metastasis, calcium gating appears to be central in the functioning of TRPM2, a 1503-amino acid channel that also gates Na^2+^ and K^+^ [24,25]. Because of its roles in proliferation and cell death, TRPM2 has been studied in several cancers, including breast, prostate, oral, neuroblastoma, and melanoma [26,27,28,29,30]. These studies demonstrated the potential importance of TRPM2 in a variety of cancers, which suggests essential roles for TRPM2 in the facilitation of cancer cell phenotypes.

A unique feature of TRPM2 is the presence of splice variants. Although the full-length TRPM2 transcript encodes for a 1503 amino acid protein [25], variants have been identified in which amino acids 538–557 are deleted (TRPM2-DN), amino acids 1291–1325 are deleted (TRPM2-DC), both the aforementioned amino acid sequences deleted (TRPM2-DNDC), and an 845 amino acid variant (TRPM2-S) [31]. In addition, two splice variant transcripts were identified via computational studies: TRPM2-TE (2138 nucleotides) and TRPM2-AS (875 nucleotides) [32]. The significance of these in relation to melanoma will be discussed later.

Another unique feature of TRPM2 is its role as one of the aforementioned chanzymes in the TRPM subfamily. TRPM2 has a C-terminal NUDT9 homology (NUDT9H) domain, which allows TRPM2 to exhibit Nudix hydrolase activity [33]. This activity catalyzes the conversion of adenosine diphosphoribose (ADP-ribose or ADPR) to adenosine monophosphate (AMP) and ribose-5-phosphate (R5P). The mechanistic importance of this enzymatic activity is that ADPR appears to activate the cation gating of TRPM2. As TRPM2 is known to induce calcium-dependent cell death in noncancerous cells following oxidative stress [34], oxidative stress stimulates the production of ADPR, which binds to the NUDT9H domain to activate the channel [33]. This results in calcium influx, which promotes calcium-mediated events such as expression of transcription factors and kinases important for sustaining cell survival and proliferation [35,36]. Through these activities, TRPM2 has been shown to regulate cell death pathways, which includes apoptosis and autophagy [34]. Since TRPM2 channels are activated by oxidative stress, peroxide concentrations have been observed to directly correlate with levels of apoptosis [37]. Because oxidative stress leads to TRPM2-mediated cell death, in noncancerous cells TRPM2 is a therapeutic target in conditions such as inflammation, stroke, and diabetes due to the protective effects elicited following TRPM2 antagonism [38,39,40]. However, these protective effects that lead to cell survival due to TRPM2 antagonism have not precluded TRPM2 from being identified as a cancer target.

### 7.1. TRPM2 Channels and Cancer

Studies in cancer cells show a paradoxical effect, where antagonism of TRPM2 selectively induces anticancer effects [30]. In fact, inhibition of TRPM2-mediated calcium influx produces altered mitochondria functionality, increased ROS production, and impairment of DNA repair processes in cancer cells [26,34]. The ultimate result of TRPM2 inhibition is decreased cell growth and proliferation in several cancers, including breast, prostate, oral, skin, and neuroblastoma [26,27,28,29,30]. It has also led to increased sensitivity to chemotherapy [41]. Evidence thus suggests that targeting TRPM2 may be a novel therapeutic approach for the treatment of several cancers.

TRPM2 cellular localization and protein expression levels appear to be unique in cancer cells. In oral cancer, increased TRPM2 expression was observed in human tongue carcinoma tissue samples and primary squamous cell carcinoma (SCC) cell lines derived from tongue carcinoma tumors [28]. Further, SCC cells exhibited greater TRPM2 ion currents when induced by ADPR. Subsequent RNAi knockdown of TRPM2 expression inhibited both growth and migration of these cells, and ultimately led to increased cell death. Regarding cellular localization, TRPM2 displays a unique subcellular localization in several types of cancers. TRPM2, which normally resides in the plasma membrane, displayed a nuclear localization in oral, breast, prostate, and skin cancer cells [26,27,28,30]. In each instance, TRPM2 was essential for growth and survival, as inhibition or RNAi knockdown led to decreased growth and increased cell death. It thus appears that the selective induction of antitumor effects in these cells is mediated through the inhibition of the nuclear role of TRPM2. To date, studies show that possible nuclear functions of TRPM2 include activation of various transcription factors, including NF-kB [42,43].

### 7.2. Multidrug Resistance

Melanoma is known to be intrinsically resistant to many typical chemotherapies and radiation [44]. The rate of this aggressive condition continues to rise, as does the public health concern regarding limited treatment options due to this resistance. Metastatic melanoma is becoming unresponsive to the therapies currently available, warranting a demand for the discovery and development of new therapies. The mechanism of resistance is multifactorial and could be caused by a defective transportation pathway, deregulation of apoptotic pathways, or changes in signaling and enzymes that communicate cell metabolic needs to the body [44]. In general, the mechanisms of resistance are poorly understood and can be dependent on factors that have not been studied or yet characterized. 

A common mechanism of resistance is through drug transport and efflux pumps, which results in decreased concentration of anti-cancer drugs in the cell targeted for treatment, thus deeming the cell line resistant. Two classes of ATP-binding cassette transporters (ABCs) actively transport the drug out of the cell and mediate the efflux that causes the decrease in drug accumulation and cell resistance, and these are P-glycoprotein (Pgp or MDR1) [45] and multidrug resistant-associated proteins (MRPs) [46]. A third drug resistance protein, the lung resistance-related protein (LRP), was identified in a lung cancer line resistant to doxorubicin treatment [47]. Each has been detected in melanoma, which has led to drug resistance in a subset of patients [48,49]. Thus, drug resistance via MDR1, MRP, or LRP has contributed to the difficulties in treating a significant cohort of melanoma patients. 

The apoptotic pathway is necessary for many cytotoxic therapies to be effective, but is known to be suppressed in melanoma, leading to drug resistance [50,51]. Apoptosis is complex, but two major pathways are well-known. The extrinsic pathway is activated by ligands leading to cell death, while the intrinsic pathway involves mitochondrial release of cytochrome c that binds to apoptotic protease activating factor-1, leading to the activated cascade resulting in cell death [52]. The survival of cells is dependent on regulator molecules such as p53, rat sarcoma (Ras), Bcl-2-family proteins, or members of the IAP family. Melanoma cell lines have been shown to possess mechanisms that confer resistance to cell death. One example is melanoma lines that contain the microRNA known as miRNA-125a, which promotes resistance to *BRAF* inhibitors and thus suppresses the intrinsic pathway of apoptosis in these cells [53]. Other examples include the overactivation of cell proliferation pathways, such as enhanced expression of the mitogen-activated protein kinase (MAPK) and AKT8 virus oncogene cellular homolog (AKT) [44]. Overexpression or increased activity of p53 also plays a role, as melanoma cell lines that contain these effects appear to be more resistant to anticancer agents such as temozolomide and fotemustine [44].

Taken together, drug resistance has contributed to the difficulties in treating certain cancers, which highlights the need for new therapies that either overcome this drug resistance or efficaciously treat patients with cancers that exhibit drug resistance. Melanoma is no exception, as drug resistance continues to be a problem in a significant amount of melanoma patients. We will subsequently provide evidence that one strategy in melanoma to overcome drug resistance, which either arises through the presence of drug efflux pumps or the evasion of apoptotic processes, is to target TRPM2.

### 7.3. Common Mutations in Melanoma

Melanoma has one of the highest rates for mutations with high variability in frequency due to exposure or absence of known carcinogens, such as UV light [54]. The Cancer Genome Atlas classifies the genome mutations that occur in melanoma into four subtypes: mutant *BRAF*, mutant *NRAS*, mutant *NF1*, and triple-wild-type [55]. Use of these subtypes can aid in predicting therapeutic options. Mutations of both driver oncogenes and single nucleotide variants are unpredictable and vary greatly between these subtypes [54]. The most common mutation is caused by chronically exposed skin to UV light, with *NF1*, *NRAS*, and *BRAF* V600K mutations involved (Table 2) [56,57,58,59]. *BRAF* V600K is less common in chronic exposure mutations but presents more frequently in intermittently exposed lesions [56]. Other lesions and tumors have presented in noncutaneous melanoma, showing that there are other causes of mutations and risks than sun exposure, such as age [56]. Patients forty years and older have been seen to have *BRAF* V600K mutations more frequently [56]. Acral lesions may include mutations to *BRAF*, *NRAS*, and *KIT* [56,57].

In melanoma, the most common mutation is in the B-raf proto-oncogene serine/threonine kinase (*BRAF*) gene. B-raf plays an essential role in activating the mitogen-activated protein kinase (MAPK)/extracellular signal related kinase (ERK) signaling pathway [56]. With this mutation, the protein is constitutively active, which leads to angiogenesis, unchecked cell replication, and the ability to metastasize [56]. Because this mutation is present in 40–50% of all melanoma patients (Table 2), B-raf inhibitors were developed, and their utilization demonstrated efficacy; however, resistance to these agents readily develops [60]. The next most common mutation in melanoma is the N-ras mutation. Ras proto-oncogenes encode a family of GDP/GTP-regulated proteins that control the growth and survival of cells [57]. Because Ras proteins are the upstream regulators of B-raf, they act as molecular switches in the signaling cascade involving ERKs. One particular member, N-ras, is mutated in 15–20% of melanoma patients [57]. This mutation results in the inability of N-ras to hydrolyze GTP into GDP. The result is the perpetual activation of downstream effectors in this pathway, which causes uncontrolled growth, migration, and metastasis [57].

In summary, melanoma is commonly known as a cancer due to driver mutations. Up to 70% of all cases appear to have either the *NRAS* or *BRAF* mutation. While the presence of these mutations leads to specific treatment options for patients, these treatment options are limited. This therefore highlights the need for additional therapies that target these driver mutations or new therapies that can overcome driver mutation effects in order to efficaciously treat melanoma patients with tumors that exhibit common mutations. We will subsequently provide evidence that one strategy for successfully treating mutant melanoma is to target TRPM2.

### 7.4. Role of TRPM2 in Melanoma

Skin cancer incidence continues to rise in the United States, where 1 in 5 people will develop this condition in their lifetime [61,62]. The three general forms of skin cancer—basal cell carcinoma (BCC), squamous cell carcinoma (SCC), and melanoma—each have an excellent prognosis if detected early [63,64,65]. The prognosis of BCC/SCC patients remains favorable as these conditions progress, but melanoma is a well-known exception. Melanoma is the most aggressive form of skin cancer, as it readily metastasizes, and the most debilitating, as it is difficult to treat in later stages [63,64]. Accordingly, malignant melanoma is the leading cause of death due to skin cancer, as it accounts for 75% of all skin cancer deaths [66]. While initial treatment for localized melanoma is surgery, several classes of systemic treatment options are available [61]. One class of agents is known as targeted therapy, where these agents are utilized based on the type of mutation present. Agents are currently available for the treatment of *BRAF*-mutation patients. However, the development of efficacious agents that target mutant Ras has proven difficult.

As previously discussed, other members of the TRPM subfamily (TRPM1, TRPM5, TRPM7, and TRPM8) appear to have essential roles in melanoma (Table 1). However, recent studies regarding TRPM2 in melanoma within the last several years have provided significant insight into its possible roles and importance in this condition. The studies appear to support the view that TRPM2 is a promising therapeutic target in melanoma.

A recent study demonstrated increased expression of TRPM2 transcripts in a primary human melanoma cell line and a human metastatic melanoma cell line [6]. Interestingly, TRPM2 transcript levels were significantly different in these cell lines, but the expression levels were above normal baseline levels. Although many cancer studies involving TRPM2 investigated Ca^2+^ influx, a unique aim of this study was to investigate the effects of K^+^ gating. As a brief review, K^+^ channels are known to have functional importance in various conditions [67,68]. Two specific K^+^ channels, the Big Potassium (BK) and calcium-activated K^+^ 3.1 (KCa3.1) channels, are voltage-gated channels activated by Ca^2+^. Activation of each causes K^+^ efflux from intracellular K^+^ stores. While it was previously demonstrated that protein kinase C potentially mediates the physiological effects induced by BK channels [69], activation of the KCa3.1 channel leads to Ca^2+^-dependent effects mediated by phospholipase C [70]. Among the physiologic functions induced by these channels are cell proliferation and cell death. Accordingly, they were previously shown to have important roles in cancer, such as colon cancer [68]. However, the results of the aforementioned melanoma study demonstrated an important role for TRPM2 in both primary and metastatic melanoma cell lines, where the gating of K^+^ was significant in facilitating melanoma progression [6]. Furthermore, this report demonstrated decreased expression levels of TRPM1. Taken together, the results demonstrated important roles for TRPM2, TRPM1, and potassium gating in the progression of melanoma.

As previously described, a unique feature of TRPM2 is the presence of splice variants. The two splice variant transcripts identified via computational studies, TRPM2-TE and TRPM2-AS, were shown to be overexpressed in melanoma [32]. Reduced levels of TRPM2-TE via stable antisense downregulation caused increased susceptibility to cell death in melanoma cells [32]. Thus, it appears that TRPM2 and its splice variants have novel roles in melanoma cells.

Our recent study reported a nuclear localization of full-length TRPM2 in three lines of human metastatic melanoma cells, while localization in noncancerous skin cells exhibited a cytoplasmic/plasma membrane localization [71]. This is in agreement with the aforementioned studies that report similar observations regarding the nuclear localization of TRPM2 in prostate, oral, and breast cancer [26,27,28]. Thus, the data from multiple types of cancers appear to support the hypothesis that TRPM2 has a unique role in cancer cells that most likely pertains to nuclear function. Because this role for TRPM2 does not appear to be applicable to noncancerous cells, antagonists of TRPM2 function are expected to selectively induce anticancer effects. Whether this nuclear role directly facilitates nuclear function or indirectly modulates nuclear events remains to be determined.

As previously mentioned, antagonism of TRPM2 function in normal versus cancerous cells was shown to elicit contrasting, but efficacious, effects in cells. TRPM2 antagonism in normal cells generally led to protective effects. More specifically, the increased cell survival due to TRPM2 inhibition led to efficacious protective effects in pancreatic b-cells, endothelial cells, and neuronal cells of the hippocampus, striatum, cortex, and substantia nigra [31]. However, in cancer cells, TRPM2 antagonism produced decreases in cell growth and increases in cell death [26,27,28,71]. These paradoxical effects were shown in a variety of cancers, which indicates a novel role for TRPM2 in these types of cancers. Currently available inhibitors utilized in these studies are shown in Table 3. Although most are non-specific and affect other targets, the antifungal agents known to inhibit TRPM2, clotrimazole, and econazole are well-known therapeutic agents currently utilized to treat human patients today. They have been utilized as experimental anticancer agents in cancer studies (including melanoma studies), and the specificity of their effects on cancer cell growth, proliferation, and cell death have been verified via TRPM2 RNAi knockdown techniques [26,27,28,71]. The search for additional TRPM2 inhibitors continues, such as those targeting the enzyme component (NUDT9H domain) of TRPM2 [72].

Accordingly, our recent study demonstrated selective anticancer effects in human metastatic melanoma cells due to the antagonism of TRPM2. TRPM2 inhibition using clotrimazole or knockdown via RNAi led to decreased growth and proliferation, and increased cell death in all melanoma lines investigated [71]. This was also in agreement with the aforementioned studies that reported similar antitumor effects due to TRPM2 antagonism in other cancers. However, our study did report two unique findings regarding the therapeutic potential of TRPM2 antagonism. First, human melanoma cell lines that express well-known drug resistance genes (*MRP1*, *LRP*) were investigated. Second, we also investigated human melanoma cell lines that harbored the B-raf or N-ras mutations. In human melanoma cell lines that contain drug resistance genes and those that exhibited all genotypes, antagonism of TRPM2 led to significant dose-dependent decreases in growth and proliferation, as well as dose-dependent increases in cell death [71]. The data thus suggested that antagonism of TRPM2 is a potential strategy to efficaciously treat melanoma patients that harbor the well-known B-raf or N-ras mutations or contain melanoma neoplasms that are drug-resistant via drug efflux pumps. Our study therefore demonstrates the potential significance of TRPM2 antagonism being used as a viable strategy to treat a wide variety of melanoma patients in the future.

## 8. Summary

In summary, several TRPM subfamily members appear to have important roles in melanoma (Table 4). In particular, recent studies have demonstrated the emerging importance of TRPM2 in melanoma. The potential of TRPM2 in melanoma treatment is based on the emerging essential role that this cation channel accomplishes in melanoma cells. The targeting of TRPM2 therefore has the potential to provide additional treatment options for melanoma patients in the future. Along with antitumor effects, TRPM2 targeting may prove to offer additional benefits, such as the prevention of melanoma. However, many questions remain to be answered. What is the nuclear function of TRPM2 in cancer cells? Does the role for TRPM2 in cancer cells entail its function as an ion channel? TRPM2 has several reported splice variants—how do these help determine its role and functions in cancer cells? Can all splice variants be targeted by drugs? The TRPs have many interactions with other proteins, including with those within the same TRPM subfamily. So in regard to its role in cancer cells, how important are potential interactions of TRPM2 with other TRPM channels? In order to establish TRPM2 as a rational therapeutic target in melanoma, it is critical that future studies resolve these questions.

## Figures and Tables

**Table 1:** Expression levels of TRPM subfamily members in skin cancers.

TRPM Channel	Expression Level	Techniques	Cell Lines	Outcome
TRPM1	Decreased	Northern blot, qPCR	B16-F1, B16-F10, CM145, HBL, 501mel, SK-Mel-28, SK-Mel-30, A2058, WM852	Increased melanoma progression, metastasis [5,6]
TRPM2	Increased	qPCR	IGR37, IGR39	Increased melanoma progression [6]
TRPM5	Increased	qPCR	B16-F1, B16-F10,	Decreased melanoma prognosis [7]
TRPM7	Increased	qPCR	B16-BL6	Tumor growth and progression? [8]
TRPM8	Increased	Immunohistochemistry	A375P, WM983A Skin biopsies	Role in SCC proliferation? [9,10]

**Table 2:** Common mutations in melanoma.

Mutation	Incidence	Features
*BRAF* V600	40–50% in cutaneous melanoma	More common in intermittently sun-exposed skin
		Causes constitutive activation of the MAP kinase/ERK signaling pathway independent of the RAS trigger
		Increased kinase activity promotes cellular growth and inhibits apoptosis
		Susceptible to *BRAF*/MEK inhibitors
*NRAS*	15–20% in cutaneous melanoma	More common in non-sun exposed skin
		Prevents GTPase activity of *NRAS*, resulting in perpetual activation
		Results in activation of MAPK, PI3K, and other pathways to promote growth and cell dysfunction
		Indicates clinically aggressive melanoma with poor prognosis
		Treatment with MEK inhibitors and/or immunotherapy; no *NRAS* inhibitors currently available
*NF1*	10–15%	High frequency in chronically sun-exposed skin
		Third most common mutation of melanoma
		Negative regulation of RAS proteins lost, resulting in activation of the RAS/RAF/MAPK pathway
		Results in cell proliferation and loss of tumor suppressor function
		Correlated with UV damage and high mutational potential; may respond to immunotherapy

**Table 3:** TRPM2 inhibitors utilized in cell studies.

TRPM2 Inhibitor	IC_50_ (TRPM2)	Other Targets
N-(p-amylcinnamoyl) anthranilic acid	1.7 µM [73]	Ca^2+^-activated Cl^−^ channels [69]
2-Aminoethoxydiphenyl borate	1 µM [74]	Store-operated Ca^2+^ gating [75]
Flufenamic acid	155.1 µM [76]	Cyclooxygenase, Cl^−^ channels, Ca^2+^-activated Cl^−^ channels, voltage-gated Ca^2+^-channels [77]
Clotrimazole	<1 µM [78]	KCa3.1 channels, cytochrome P-450 enzymes [6,79]
Econazole	<1 µM [78]	Store-operated Ca^2+^ gating, phosphatidylinositol-3-kinase (PI3K) [80,81]

**Table 4:** Summary of roles of TRPM subfamily members in melanoma.

TRPM Channel	Potential Role(s)
TRPM1	Tumor suppressor, prognostic biomarker
TRPM2	Tumor growth and progression, cell death
TRPM3	Biomarker for MAR
TRPM4	Prognostic biomarker for UVM
TRPM5	Prognostic biomarker for highly aggressive melanoma
TRPM7	Tumor growth and progression
TRPM8	Tumor growth and progression

## Data Availability

No new data were created or analyzed in this study. Data sharing is not applicable to this article.
